# Evaluating Processing Speed and Reaction Time Outcome Measures in Children and Adolescents with Down Syndrome

**DOI:** 10.3390/ijerph20065202

**Published:** 2023-03-15

**Authors:** Emily K. Schworer, Mekibib Altaye, Deborah J. Fidler, Dean W. Beebe, Susan Wiley, Emily K. Hoffman, Anna J. Esbensen

**Affiliations:** 1 Division of Developmental and Behavioral Pediatrics, Cincinnati Children’s Hospital Medical Center, Cincinnati, OH 45229, USA; 2 Waisman Center, University of Wisconsin-Madison, Madison, WI 53705, USA; 3 Department of Pediatrics, University of Cincinnati College of Medicine, Cincinnati, OH 45267, USA; 4 Division of Biostatistics and Epidemiology, Cincinnati Children’s Hospital, Cincinnati, OH 45229, USA; 5 Human Development and Family Studies, Colorado State University, Fort Collins, CO 80523, USA; 6 Division of Behavioral Medicine and Clinical Psychology, Cincinnati Children’s Hospital Medical Center, Cincinnati, OH 45229, USA

**Keywords:** Down syndrome, processing speed, outcome measures, cognition, clinical trials

## Abstract

Reliable and valid cognitive outcome measures, including examiner-administered and computer-facilitated assessments of processing speed and reaction time, are necessary for future clinical trials that include individuals with Down syndrome (DS). The current study evaluated the score distributions and psychometric properties of four examiner-administered and three computerized processing speed and reaction time measures. Participants included 97 individuals with DS, aged 6 to 17 (M = 12.6, SD = 3.3). Two examiner-administered measures (Differential Ability Scales-II Rapid Naming and Cat/dog Stroop Congruent) met most predetermined psychometric criteria. Other assessments demonstrated good test-retest reliability and had negligible practice effects but lacked adequate feasibility. Recommendations for using processing speed and reaction time assessments in research and suggestions for modifications of measures are discussed.

## 1. Introduction

Common cognitive features of individuals with Down syndrome (DS) include challenges with executive function, learning, and memory [1,2,3,4,5,6,7]. Two related cognitive components that impact executive function abilities are processing speed, or the efficiency of cognitive task completion, and reaction time [8,9]. Processing speed and reaction time are components of the Cattell-Horn-Carroll Theory of cognitive abilities and can be assessed through measurement of the fluency of completing a simple task (Gs) or reaction time (Gt) [10]. These types of measures have been used to assess outcomes in previous interventions and pharmaceutical studies in DS [11,12]. Because processing speed and reaction time are underlying cognitive components in numerous cognitive processes, it is a domain that continues to be used to assess cognitive outcomes in clinical trials and intervention research involving individuals with DS.

### 1.1. Processing Speed in Down Syndrome

There is substantial evidence for difficulties with processing speed and reaction time throughout the lifespan in DS, with an emphasis on challenges in this area in adulthood [13,14,15]. Despite the focus on older individuals, significant differences in processing speed components, such as strategy production and completion time, have been observed in infants with DS relative to chronological age-matched typically developing infants [14]. There is also evidence that processing speed and reaction time are slower in children, adolescents, and adults with DS compared to same-age peers with typical development and intellectual disability [13,15,16,17,18]. Within syndrome differences have also been reported based on verbal or visuospatial cueing used in processing speed and reaction time measures [19].

Although there is evidence of challenges with processing speed and reaction time specific to DS, findings related to this cognitive domain have been mixed. For example, similar performance has been found between reaction times in individuals with DS compared to peers with intellectual disability of other etiologies [20]. This suggests the slower reaction time in DS may not necessarily be specific to the behavioral phenotype associated with DS. Another study also found that multiple processing speed measures (line comparison, pattern comparison, and reproduction of simple shapes) were not significantly different between children and adolescents with DS compared to children with typical development matched on verbal mental age [21]. Additionally, there is likely overlap between processing speed and other challenges in DS, primarily motor abilities and responses [14,22,23,24]. Regardless of motor contributions to presentation and the specificity of processing speed and reaction time challenges to the DS phenotype, evidence for difficulties with processing speed and reaction time in DS compared to same-age typically developing peers is likely to lead to use of this cognitive domain as a treatment outcome in future clinical trials.

### 1.2. Clinical Trial Preparation for Down Syndrome

As new advances in interventions specific to DS continue to be made, reliable and valid outcome measurements are of critical importance in clinical trials to determine intervention effectiveness. NIH INCLUDE (INvestigation of Co-occurring conditions across the Lifespan to Understand Down syndromE) initiatives support the need for evaluation of measures to ensure they are feasible and psychometrically sound for individuals with DS [25,26]. Although norming procedures for standardized measures often include a small portion of individuals with learning disabilities or intellectual disabilities, norms are primarily developed based on the performance of individuals with typical development. This limited representation leads to potential challenges with floor effects or restricted range in the performance of individuals who have lower overall cognitive abilities and a subsequent dearth in available outcome measures for intervention work in this population. Progress has been made in evaluating cognitive outcome measures for DS [27,28,29,30]. However, processing speed and reaction time have undergone limited measurement evaluation for individuals with DS, despite the importance of the construct. 

### 1.3. Processing Speed and Reaction Time Measurement

Indicators of processing speed assess the amount of time to complete tasks or the efficiency (i.e., number of correct responses) of task completion within a set time while also considering the accuracy of participant responses. This is also what is referred to as Gs in the Cattell-Horn-Carroll Theory of cognitive abilities [10]. Among individuals with DS, tasks within the Rate of Test Taking (R9) of Gs have been evaluated. One example of this type of task is the Cat/dog Stroop task, a modified version of the Day-night Stroop task [31]. Although inhibition can be assessed with the number of errors, an additional timed aspect of the task also taps processing speed and has been used in studies that include adults with DS [32,33]. Despite the successful use of these measures in adults with DS, given the potential impact of developmental differences, it is necessary to assess the utility of the Cat/dog Stroop task in children and adolescents with DS. 

There are also processing speed (Gs) subtests of larger clinical assessments. Rapid Naming is a processing speed measure that is part of the Differential Ability Scales, second edition (DAS-II) and involves labeling colors and animals in timed tasks. The DAS-II Rapid Naming subtest was designated as promising by NIH’s DS outcome measure working group [26] and a similar color-shape naming task has been used as an outcome measure in a safety and efficacy pharmaceutical trial in DS [12]. Other timed clinical assessments can be used as indicators of processing speed, such as cancellation tasks. These types of tasks have been used to assess cognition in early-stage Alzheimer’s disease for adults with DS [34,35] and to monitor outcomes in a safety and efficacy pharmaceutical trial for 10- to 17-year-olds with DS [36]. Cancellation tasks are determined by experts to be promising for individuals with DS, especially considering the limited language requirements of these tasks, but no psychometric evaluations of cancellation measures have been completed to date [26].

Reaction time can be assessed using a variety of methods as an indicator of Gt in the Cattell-Horn-Carroll Theory of cognitive abilities [22,37]. Only a small number of Simple Reaction Time (R1) tasks have been evaluated in children and adolescents with DS or other neurogenetic disorders; however, evaluated measures show promising feasibility and score distributions [10]. For example, the Cambridge Neuropsychological Test Automated Battery (CANTAB) Simple Reaction Time had minimal floor effects (9%) in children and adults with DS [29]. The Test of Attentional Performance for Children (KiTAP) Alertness subtest is another measure of reaction time, which requires participants to tap a button when a stimulus appears on the computer screen. Evaluations of the KiTAP Alertness in Fragile X syndrome (FXS) provide evidence for the utility of the measure with individuals with developmental disabilities [38]. 

### 1.4. Current Study

To prepare for clinical trials, there is a significant need for the evaluation of processing speed and reaction time measures in children and adolescents with DS. Both examiner-administered processing speed (Gs; DAS-II Rapid Naming, Wechsler Intelligence Scale for Children-V Cancellation, Leiter-3 Attention Sustained, and Cat/dog Stroop) and computer-facilitated reaction time (Gt; CANTAB Reaction Time, KiTAP Alertness, and Conners Continuous Performance Test-3) assessments were evaluated for their appropriateness for use in research involving children and adolescents with DS. The current study aimed to evaluate the feasibility of processing speed and reaction time measures, with specific investigation of floor effects and score distributions. The second goal of the study was to evaluate the psychometrics of the processing speed and reaction time measures (i.e., practice effects, test-retest reliability, and validity). Post hoc analyses were also completed for processing speed and reaction time measures that fell below feasibility criterion to describe the cognitive abilities and age of individuals who were able and unable to complete the tasks. 

## 2. Materials and Methods

### 2.1. Participants

Participants were recruited from two US cities through outreach to DS associations and other local organizations. The study included 97 individuals with DS, aged 6 to 17 years (M = 12.6, SD = 3.3). The average IQ for the sample was 49.1, SD = 5.4, and ranged from 47 to 76 on the Stanford Binet, fifth edition abbreviated battery. Fifty of the participants were male (51.5%) and 47 were female (48.5%). Race and ethnicity were predominately White (85.6%) and non-Hispanic (94.8%). Other participants were Black (5.2%), Asian (5.2%), and other/mixed race (4.0%). Subsets of the participants in the current study were also included in studies investigating working memory, social cognition, short-term memory, and verbal fluency outcome measures [27,28,39,40].

### 2.2. Procedure

All study procedures were approved using the Single IRB platform at Cincinnati Children’s Hospital Medical Center. Children and adolescents with DS participated in two study visits. The second visit occurred approximately two weeks after the first. During both visits (Time 1 and Time 2), participants completed seven processing speed/reaction time tasks, as part of a larger set of clinical assessments of cognition and language. Caregivers completed a questionnaire rating their child’s adaptive behavior. Each study session was approximately 1.5–2.5 h. Tasks were randomized into blocks to limit the impact of attention or fatigue on performance.

### 2.3. Measures

#### 2.3.1. Measures Evaluated

##### Processing Speed (Gs)

Key processing speed measures were selected that have been described by publishers as processing speed assessments or those that place considerable demand on the speed of performance on relatively simple cognitive tasks. Assessments include a variety of types of measures within the Rate of Test Taking (R9) processing speed tasks (Gs). Measure selection was informed by the NIH working group recommendations [26], and attention to task demands that are appropriate for children and adolescents with DS. All measures were designed or normed for the ages of the participants in the current study unless otherwise specified. Standard administration was followed and is described below for each measure.

1. Differential Ability Scales, Second Edition (DAS-II) Rapid Naming.

The DAS-II Rapid Naming measures fluency in responses to visual stimuli [41]. This measure included a color, animal, and combined component that each consisted of 35 pictures. A short untimed practice trial was completed before each component. The number correct and time for participants to label each set (maximum time 120 s per set) were used for scoring. Total scores for all three components of the measure generated an overall ability score (analogous to raw scores) and T-score, with higher scores representing better processing speed.

2. Wechsler Intelligence Scale for Children, Fifth Edition (WISC-V) Cancellation.

The WISC-V Cancellation assesses processing speed and visual discrimination [42]. The participant was required to scan images in a workbook and mark the animals. Images were organized randomly for the first trial and structured (i.e., in straight rows) for the second trial. Participants had 45 s to complete each trial. Before the first trial, practice was administered, and teaching was provided by the examiner as needed. If the child’s motor abilities did not include pencil/crayon use, participants pointed to the images and the examiner marked the page. The number of errors was subtracted from the total number of correctly marked animals and the random and structured tasks were combined for a total raw score. The WISC-V Cancellation total raw and scaled scores were included in the current study, and high scores reflect better processing performance.

3. Leiter International Performance Scale, Third Edition (Leiter-3) Attention Sustained.

The Leiter-3 Attention Sustained is a task of processing speed, visual discrimination, and fluency [43]. A target picture was displayed at the top of the page (e.g., a triangle). The participant was required to mark images that matched the target stimuli and images became more visually complex across trials. There were four trials, with two lasting 30 s and two lasting 60 s. All trials included practice and opportunities for teaching. If motor abilities of the participant did not include pencil/crayon use, participants pointed to the images and the examiner marked the page. A general evaluation of the Leiter-3 found minimal floor effects in adolescents and adults with DS [30]. The number of errors was subtracted from the total number of correctly marked target images. The present study included raw and scaled scores, and higher scores indicate better performance.

4. Cat/dog Stroop.

The Cat/dog Stroop task, originally developed as a measure of inhibition [31], can also assess processing speed by examining performance times for trials individually. Participants were first required to label cats and dogs on a grid of stimuli with 16 pictures (8 cats and 8 dogs). After the congruent trial, participants named cats as “dogs” and dogs as “cats.” Two separate practice/training trials were administered prior to the congruent and incongruent tests. This task is a modification of the common Day/night Stroop task and has been implemented in research involving adults with DS [31,32,33]. Completion times in seconds for the congruent and incongruent labeling tasks were evaluated in the current study, as standardized scoring that accounts for accuracy is not available, and higher scores represent slower processing speeds.

##### Reaction Time (Gt)

Key reaction time measures were selected that have been described by publishers as reaction time assessments on relatively simple cognitive tasks. Assessments include a variety of types of measures within Simple Reaction Time (R1) tasks (Gt). Measure selection was performed by using the NIH working group recommendations [26], and the task demands that are appropriate for children and adolescents with DS were considered. All measures were designed or normed for the ages of the participants in the current study unless otherwise specified. Standard administration was followed and is described below for each measure.

1. Cambridge Neuropsychological Test Automated Battery (CANTAB) Reaction Time (RTI).

The CANTAB RTI assesses cognitive and motor responses through reaction time using a tablet activity [44]. Participants were required to hold down a circle icon at the bottom of the screen. While holding the circle icon, another circle icon flashed yellow near the top of the screen, indicating it was time to release the first circle and tap the circle that flashed yellow. Participants were encouraged to move their finger as quickly as possible. A previous version of the task, the SRT, has shown good feasibility [29]; however, the SRT is no longer available and the current version (RTI) has not been evaluated in DS. Variables included in the current study were the simple median movement time (i.e., median time in milliseconds for a participant to release the bottom button and select the circle icon that flashed yellow) and simple median reaction time (i.e., median duration in milliseconds for a participant to release the bottom button after the circle icon flashes yellow). Lower scores indicate faster reaction time.

2. Test of Attentional Performance for Children (KiTAP) Alertness.

The KiTAP Alertness task measures the reaction time and information processing speed [45]. The KiTAP was administered on a laptop computer with an externally connected response button. The screen displayed a castle-themed stimulus on the screen, and the participant was required to tap the response button as quickly as possible. The KiTAP Alertness reaction time variable showed high feasibility and reliability in individuals with FXS and other clinical groups [38,46]. The median reaction time was evaluated in the current study, and lower scores reflect faster reaction time.

3. Conners Continuous Performance Test, Third Edition (CPT-3).

The Conners CPT-3 is a measure of attentiveness and impulsivity [47]. Participants responded (by hitting the space bar) when all letters, except for the letter “X”, were displayed on a computer screen. This measure has been used previously in studies that include children with attention deficit hyperactivity disorder [48,49]. Because the task is designed for children 8 years and older [47], 6- and 7-year-olds in the current study were not administered the task. The Conners CPT-3 Hit reaction time (HRT) T-score was used as an indicator of reaction time for the current study, and lower scores indicate faster reaction time. 

#### 2.3.2. Descriptive Measures

##### Adaptive Behavior

The Vineland Adaptive Behavior Scale, Third Edition (VABS-3) [50] was used to assess adaptive skills and was completed by caregivers. Social, daily living, and communication skills are measured using the VABS-3 to determine an Adaptive Behavior Composite (ABC). The VABS-3 ABC standard score (M = 100, SD = 15) was used to compare processing speed and reaction time measures to overall adaptive behavior. 

##### Cognitive Abilities

The Stanford Binet, Fifth Edition (SB-5) abbreviated battery IQ (ABIQ) was used to assess general verbal and nonverbal cognitive abilities [51]. Deviation scores were obtained from SB-5 online scoring software (https://www.proedinc.com/Products/14462/sb5-online-scoring-and-report-system-1-year-base-subscription-includes-5-licenses.aspx) to eliminate floor effects [52]. Standard ABIQ scoring was used to describe the sample and deviation ABIQ scores were compared to processing speed and reaction time measures. 

##### Vocabulary

Expressive vocabulary was assessed using the Expressive Vocabulary Test, Second and Third Editions (EVT-2 and EVT-3) [53,54]. The measure is normed for children and adults and has been deemed appropriate for use in DS by experts [26]. Expressive vocabulary standard scores (M = 100, SD = 15) were compared to processing speed and reaction time measures. The majority of participants were administered the EVT-3, n = 81. Receptive vocabulary was measured with the Peabody Picture Vocabulary Test, Fourth and Fifth Editions (PPVT-4 and PPVT-5) [55,56]. This assessment is normed for children and adults and is appropriate for research in DS [26]. Receptive vocabulary standard scores (M = 100, SD = 15) were compared to processing speed and reaction time measures. The majority of participants were administered the PPVT-5, n = 81.

#### 2.3.3. Data Analysis

Each of the processing speed and reaction time measures was evaluated based on feasibility, score distributions, practice effects, test-retest reliability, validity, and sensitivity/specificity analyses when appropriate. The feasibility (i.e., the percentage of participants who completed/generated correct or incorrect responses at both visits) was assessed for the examiner-administered processing speed and computer-facilitated reaction time measures. Predetermined criterion for feasibility was set at ≥80% based on acceptable feasibility used in previous studies [28,57]. Examiners selected a reason for non-completion for participants that did not generate responses for a particular task. Next, score distributions were examined through inspection of means, medians, skewness, and kurtosis. The acceptable range of skewness was between −1 and 1 and kurtosis was between −2 and 2. Floor effects were also examined and defined as the sum of the number of participants who completed but received the lowest score on a task at Time 1 and the number of participants who were not able to complete or generate responses on the task. Criterion for acceptable floor effects was set at less than 20% [28]. 

The second aim of the study was to evaluate the psychometrics of the processing speed and reaction time measures (i.e., practice effects, test-retest reliability, and convergent validity). Paired samples *t*-tests and effect sizes were used to investigate practice effects. Practice effects were considered problematic if there were significant differences between Time 1 and Time 2 and Cohen’s *d* was greater than 0.20 [28]. Intraclass correlation coefficients (ICC) were calculated to evaluate test-retest reliability and included categories of poor (<0.50), moderate (0.50–0.74), good (0.75–0.90), or excellent (>0.90) [58]. Test-rest reliability was considered acceptable if ICCs were at or above 0.75 (i.e., good or excellent categories). Spearman correlations were used to assess convergent validity among the raw/ability scores of the processing speed and reaction time measures and associations ≥ 0.50 were considered adequate evidence of validity among measures. Convergent validity was not assessed for the T-scores/scaled scores due to significant floor effects on multiple measures. Associations between Time 1 processing speed/reaction time and age, adaptive behaviors, cognitive abilities, and vocabulary were also evaluated using Spearman correlations.

Finally, post hoc sensitivity and specificity were analyzed for measures with feasibility between 20% and 80%. Measures below 20% feasibility were not considered, as such few participants were able to complete the task. Sensitivity ratios describe participants in the study sample who were accurately identified as able to complete a measure. Specificity ratios describe the participants in the study sample who were accurately identified as unable to complete a measure. Benchmarks of age (8 and 10 years) and cognitive abilities (no restriction, deviation ABIQ ≥ 30, 40, and 50) were investigated to determine participant characteristics that increased the probabilities of completion of a measure. Benchmarks were selected based on previous investigations of outcome measure sensitivity and specificity and the ages of children in clinical trials [12,27,28].

## 3. Results

### 3.1. Study Aim 1: Feasibility and Floor Effects 

Table 1 presents a summary of the median, range, skewness, kurtosis, feasibility, and floor effects for the processing speed and reaction time measures at Time 1. Of the seven examiner-administered processing speed and computer-facilitated reaction time assessments evaluated in the current study, two met predetermined feasibility criterion (>80%). Feasibility for the DAS-II Rapid Naming task was 86.6%, and the Cat/dog Stroop Congruent was 91.8%. Other assessments (WISC-V Cancellation, Leiter-3 Attention Sustained, and Cat/dog Stroop Incongruent) had moderate feasibility (70.1–71.1%). Reasons for non-completion for the WISC-V Cancellation and Leiter-3 Attention Sustained were similar. These included not understanding instructions (14%, 16%), noncompliance (3%, 4%), verbal refusal (1%, 1%), participant fatigue (1%, 1%), and only completing at one visit (11%, 8%). For the Cat/dog Stroop Incongruent task, reasons for not completing the task were not understanding instructions (13%), limited verbal ability (3%), noncompliance (2%), verbal refusal (1%), and only completing at one visit (10%).

Computer-facilitated reaction time assessments (CANTAB RTI, KiTAP Alertness, and Conners CPT-3) had low feasibility (8.6–52.1%). For the CANTAB RTI, reasons for non-completion were not understanding instructions (26%), noncompliance (8%), verbal refusal (5%), participant fatigue (10%), and only completing at one visit (4%). For the KiTAP Alertness, reasons for non-completion were not understanding instructions (15%), noncompliance (6%), verbal refusal (3%), participant fatigue (14%), acquiescence (i.e., responding without attending to stimuli; 1%), and only completing at one visit (9%). The majority of participants were not able to complete the Conners CPT-3, and reasons for non-completion included not understanding instructions (49%), noncompliance (5%), verbal refusal (5%), participant fatigue (18%), acquiescence (6%), and only completing at one visit (10%). 

Skew and kurtosis were high for Cat/dog Stroop and the CANTAB RTI. Skew was also high for WISC-V Cancellation scaled score, Leiter-3 Attention Sustained scaled score, and KiTAP Alertness. Floor effects were unacceptable (>20%) for all measures, except for the DAS-II Rapid Naming and Cat/dog Stroop Congruent time (see Table 1). Floor effects were more problematic for standardized scores compared to raw scores for the WISC-V Cancellation and Leiter-3 Attention Sustained. Due to the markedly low feasibility of the Conners CPT-3 (8.6%), no further analyses were completed for this measure.

### 3.2. Study Aim 2: Practice Effects, Test-Retest Reliability, and Validity 

Practice effects were not identified for any of the processing speed and reaction time measures, with no significant differences between performance at Time 1 and Time 2 and Cohen’s *d* ≤ 0.20 (Table 2). Test-retest reliability was good for the majority of the measures (DAS-II Rapid Naming ability and T-Scores, WISC-V Cancellation total raw and scaled scores, Leiter-3 Attention Sustained total raw and scaled scores, CANTAB RTI simple median reaction time, and KiTAP Alertness median reaction time). The CANTAB RTI simple median movement time and Cat/dog Stroop Congruent time had moderate test-retest reliability (ICC = 0.70; 0.65), and the Cat/dog Stroop Incongruent time had poor reliability (ICC = 0.46). 

There were multiple significant correlations among the processing speed and reaction time measures and several of the measures showed evidence of convergent validity (*r* ≥ 0.50; Table 3). First, there were significant associations between the DAS-II Rapid Naming ability score and Cat/dog Stroop Congruent/Incongruent time (*r* = −0.67, −0.66). The WISC-V Cancellation total raw score was also significantly correlated with the Leiter-3 Attention Sustained total raw score (*r* = 0.75), Cat/dog Stroop Congruent time (*r* = −0.59), and KiTAP Alertness median reaction time (*r* = −0.56). Additionally, the Leiter-3 Attention Sustained total raw score was correlated with the Cat/dog Stroop Congruent time (*r* = −0.52). The CANTAB RTI simple median reaction time was also correlated with the KiTAP Alertness median reaction time (*r* = 0.59).

Correlations with broader developmental domains were identified between processing speed and reaction time measures and age, adaptive behaviors, cognitive abilities, and vocabulary (Table 2). Age was significantly correlated with the DAS-II Rapid Naming ability score, WISC-V Cancellation total raw score, Leiter-3 Attention Sustained total raw and scaled scores, Cat/dog Stroop Congruent and Incongruent time, and KiTAP Alertness median reaction time. Adaptive behaviors (VABS-3 ABC) were significantly associated with all measures except the Cat/dog Stroop Congruent time and computer/tablet tasks (CANTAB RTI and KiTAP Alertness). Cognitive abilities were significantly correlated with the DAS-II Rapid Naming T-Score, Leiter-3 Attention Sustained total scaled score, and CANTAB RTI simple median movement time. Both expressive and receptive vocabulary were significantly associated with DAS-II Rapid Naming T-Score, WISC-V Cancellation total raw and scaled scores, Leiter-3 Attention Sustained total scaled score, Cat/dog Stroop Congruent time, and CANTAB RTI simple median movement time.

### 3.3. Study Aim 3: Post Hoc Completion Sensitivity and Specificity 

Sensitivity and specificity ratios were computed for the WISC-V Cancellation, Leiter-3 Sustained Attention, Cat/dog Stroop Incongruent, CANTAB RTI, and KiTAP Alertness, as they did not meet predetermined criterion for feasibility (Table 4 and Table 5). Similar ratios were reported for benchmark ages 8 and 10. As cognitive ability benchmarks increased, sensitivity decreased, and specificity increased. A balance of moderately high sensitivity and specificity was greatest for ABIQ Deviation ≥ 30 and age greater than 8 for the examiner-administered processing speed tasks (Table 4) and ABIQ Deviation ≥ 30 and age greater than 10 for the computer-facilitated reaction time tasks (Table 5).

## 4. Discussion

This study evaluated examiner-administered assessments of processing speed and computer-facilitated assessments of reaction time in children and adolescents with DS. The DAS-II Rapid Naming and Cat/dog Stroop Congruent tasks were feasible and had good or adequate psychometrics. Other standardized measures (WISC-V Cancellation and Leiter-3 Attention Sustained) had good test-retest reliability, negligible practice effects, and some convergent validity, despite having only moderate feasibility. Computer-facilitated tasks had low feasibility and the computerized measures selected for evaluation may not be appropriate for individuals with DS. A summary of measures that met/did not meet study criteria is presented in Table 6.

### 4.1. Feasibility and Floor Effects 

Two measures met predetermined study criterion for feasibility, the DAS-II Rapid Naming and Cat/dog Stroop Congruent time. Although both tasks required verbal abilities, which could be a barrier to completion for some participants, the majority of the sample was able to complete these tasks. The WISC-V Cancellation, Leiter-3 Attention Sustained, and Cat/dog Stroop Incongruent time did not reach adequate feasibility criterion; however, they were approaching acceptable feasibility at approximately 70%. Because these tasks were completed by many participants, it is recommended that they be used with caution in future research, with specific monitoring of the motor components of the WISC-V Cancellation and Leiter-3 Attention Sustained tasks. It is also possible that additional training, practice trials, or simplification of measures would increase feasibility. For example, the WISC-V Cancellation could be modified to have larger pictures or a matrix with fewer pictures and standardized for individuals with DS and other intellectual and developmental disabilities. Modifying cancellation tasks to make them more accessible would be beneficial, as visuospatial assessment modalities are needed to support inclusion of minimally verbal individuals with DS in clinical trials. 

The evaluated computer-facilitated reaction time measures (CANTAB RTI, KiTAP Alertness, and Conners CPT-3) all had low feasibility. This suggests that the selected measures were not a good match for the cognitive abilities of the children and adolescents with DS in the current study. Previous findings on feasibility and floor effects on computer-facilitated measures have been mixed [27,29], and results from the current study add to the uncertainty of the utility of specific computerized cognitive assessments. The lowest feasibility was found for the Conners CPT-3, and future studies should explore the Kiddie CPT-3 that is available for children with a younger mental age, as it is likely the nature of the task rather than computer presentation was impacting feasibility. 

Floor effects corresponded with feasibility results for raw/ability processing speed scores and reaction times. For all standardized measures, floor effects were greater for standard scores than raw and ability scores. This was particularly apparent for the WISC-V Cancellation and Leiter-3 Attention Sustained scaled scores, and thus, raw scores should be used when scoring these measures for individuals with DS. Additional development of age-corrected standard scores for individuals with intellectual disabilities would be helpful to allow for some form of age correction in scores for these measures. 

### 4.2. Practice Effects, Test-Retest Reliability, and Validity 

Overall, practice effects were negligible for all evaluated processing speed and reaction time measures, and thus, scores did not improve over the two-week testing period. Test-retest reliability was also good for all processing speed measures, except for Cat/dog Stroop and CANTAB RTI simple median movement time. There was also some evidence of convergent validity among processing speed and reaction time measures (see Table 3 and Table 6). Notably, both cancellation tasks (WISC-V Cancellation and Leiter-3 Attention Sustained total raw scores) were positively correlated. The DAS-II Rapid Naming ability score was also negatively correlated with the Cat/dog Stroop scores, such that as the Rapid Naming scores increased, the time to complete the congruent and incongruent Cat/dog Stroop task decreased (i.e., quicker processing speed). 

Correlations between the processing speed and reaction time measures and broader developmental domains were also observed. Most of the correlations with age were in the expected direction, such that as performance improved (i.e., higher or faster scores), age also increased. Although this study was cross-sectional, the correlations with age suggest individuals with DS may make age-related gains on measures of processing speed and additional investigation of processing speed measures in adults with DS will be beneficial for evaluating the utility of these measures across the lifespan. For example, the Cat/dog Stroop has been used successfully in groups of adults with DS in previous research [31,33], and therefore, it may be a good measure to use if a study includes a wide participant age range.

Many of the examiner-administered processing speed assessments were moderately associated with adaptive behavior. Fewer processing speed and one reaction time measures were correlated with cognitive abilities (DAS-II Rapid Naming T-score, Leiter-3 Attention Sustained scaled score, and CANTAB RTI simple median movement time), suggesting that the processing speed abilities and reaction time are separate from overall cognition. The EVT and PPVT were also significantly associated with some processing speed and reaction time measures that required both verbal and nonverbal responses. Correlations between expressive vocabulary and processing speed measures that did not require a verbal response (WISC-V Cancellation, Leiter-3 Attention Sustained, and CANTAB RTI) were unexpected and warrants additional investigation to better characterize the relation between verbal skills and processing speed and reaction time performance. 

### 4.3. Post Hoc Completion Sensitivity and Specificity

Examiner-administered processing speed and computer-facilitated reaction time measures both had relatively high sensitivity and specificity for ABIQ Deviation ≥ 30. There were differences based on age, with the computer-facilitated reaction time measures having higher sensitivity and specificity for the age 10 benchmark, indicating that computerized tasks may be better suited for older individuals with DS. These findings should be considered for use as guidelines for inclusion criteria in DS clinical trials if specific measures are required for tracking treatment outcomes. Even with these guidelines, there remains a need to develop measures that are feasible for a wider range of processing speed and reaction time abilities to accommodate the cognitive heterogeneity observed in individuals with DS [59]. 

### 4.4. Limitations and Future Directions

In this evaluation of processing speed, study limitations should be considered in the interpretation of results. First, processing speed tasks invariably also call upon other skills (“task impurity”) [60]. For example, in addition to processing speed, the WISC-V Cancellation or Leiter-3 Attention Sustained tasks require some degree of inhibitory control and working memory (i.e., inhibiting the selection of incorrect items and remembering the rules for which target stimuli to select). There is also a need for longitudinal studies of measures with good feasibility that simulate the timeline of clinical trials to better understand how performance changes throughout development. Computer-facilitated reaction time assessments were also always completed after the examiner-facilitated assessment battery and should be randomized in future investigations of these types of measures. Modifications or additional measurement development should also be explored in future studies to provide more options for processing speed and reaction time outcome measures for all individuals with DS, especially children and adolescents with lower cognitive or language abilities. 

## 5. Conclusions

The DAS-II Rapid Naming and Cat/dog Stroop Congruent tasks are recommended as assessments of processing speed for use in future clinical trials. Because these measures both have a verbal component, there remains a need to identify a nonverbal processing speed measure with high feasibility for children and adolescents with DS. Other measures of processing speed require modification (Cat/dog Stroop Incongruent, WISC-V Cancellation, and Leiter-3 Attention Sustained), but could still be useful measures for this population as the feasibility was moderate and other psychometric properties were generally good. Adjustments to computer-facilitated reaction time measures are needed to make these assessments accessible to children and adolescents with DS. These findings contribute to the necessary evaluation of cognitive measures in DS and will serve as an important guide for outcome measure selection in future clinical trials that include individuals with DS.

## Figures and Tables

**Table 1:** Processing speed and reaction time performance, feasibility, and floor effects at Time 1.

	Median (Range)	Skew	Kurtosis	Feasibility n (%)	Participants at Floor n (%) ^a^
DAS-II Rapid Naming Ability Score	123 (54–172)	−0.29	−0.34	84 (86.6%)	13/97 (13.4%)
DAS-II Rapid Naming T-Score Score	32 (10–47)	−0.30	−0.55		14/97 (14.4%)
WISC-V Cancellation Total Raw Score	31 (−16–74)	0.21	0.37	68 (70.1%)	29/97 (29.9%)
WISC-V Cancellation Scaled Score	1 (1–9)	1.46	0.91		69/97 (71.1%)
Leiter-3 Attention Sustained Total Raw Score	46 (−11–109)	0.30	0.11	68 (70.1%)	29/97 (29.9%)
Leiter-3 Attention Sustained Scaled Score	0 (0–8)	1.37	0.74		71/97 (73.2%)
Cat/dog Stroop Congruent Time (s)	14 (8–126)	5.87	43.90	89 (91.8%)	8/97 (8.2%)
Cat/dog Stroop Incongruent Time (s)	23 (11–101)	3.28	16.39	69 (71.1%)	28/97 (28.9%)
CANTAB RTI Simple Median Reaction Time (ms)	410 (285–1079)	2.02	4.66	44 (47.3%)	49/93 ^b^ (52.7%)
CANTAB RTI Simple Median Movement Time (ms)	335 (28–1015)	1.93	8.23		49/93 ^b^ (52.7%)
KiTAP Alertness Median Reaction Time (ms)	614 (280–2020)	1.27	1.39	49 (52.1%)	45/94 ^b^ (47.9%)
Conners CPT-3 HRT T-score	62 (41–90)	0.53	−0.81	7 (8.6%)	77/81 ^bc^ (95.1%)

^a^ Participants at floor included participants with the lowest score on the measure and those unable to complete the measure; ^b^ Technology error removed from feasibility and floor denominator; ^c^ Conners CPT-3 not administered to children 6 and 7 years old; DAS-II = Differential Ability Scales, Second Edition; WISC-V = Wechsler Intelligence Scale for Children, Fifth Edition; Leiter-3 = Leiter International Performance Scale, Third Edition; CANTAB RTI = Cambridge Neuropsychological Test Automated Battery Reaction Time; KiTAP = Test of Attentional Performance for Children; CPT-3 HRT = Continuous Performance Test, Third Edition Hit Reaction Time.

**Table 2:** Practice effects, test-retest reliability, and associations with broader developmental domains.

	Time 1 Mean (SD)	Time 2 Mean (SD)	Mean Difference	Cohen’s *d*	ICC	Age	VABS-3 ABC	SB-5 ABIQ ^a^	EVT	PPVT
DAS-II Rapid Naming Ability Score	123.87 (24.72)	122.80 (27.05)	1.07	0.08	0.85	0.52 ***	0.25 *	0.17	0.20	0.21
DAS-II Rapid Naming T-Score	31.06 (8.78)	30.83 (9.44)	0.23	0.04	0.84	−0.08	0.40 ***	0.42 ***	0.44 ***	0.48 ***
WISC-V Cancellation Total Raw Score	34.41 (16.59)	35.88 (16.85)	−1.47	−0.15	0.84	0.31 **	0.35 **	0.17	0.28 *	0.40 ***
WISC-V Cancellation Scaled Score	2.68 (2.46)	2.94 (2.65)	−0.26	−0.19	0.85	−0.18	0.35 **	0.17	0.41 ***	0.51 ***
Leiter-3 Attention Sustained Total Raw Score	51.37 (25.16)	53.26 (29.79)	−1.90	−0.11	0.81	0.32 **	0.25 *	0.23	0.14	0.29 *
Leiter-3 Attention Sustained Scaled Score	1.44 (2.19)	1.63 (2.46)	−0.19	−0.12	0.78	−0.41 ***	0.24 *	0.33 **	0.34 **	0.43 ***
Cat/dog Stroop Congruent Time (s)	17.71 (13.47)	16.99 (8.53)	0.72	0.07	0.65	−0.45 ***	−0.19	−0.19	−0.22 *	−0.22 *
Cat/dog Stroop Incongruent Time (s)	23.98 (9.09)	24.56 (9.65)	−0.57	−0.06	0.46	−0.38 **	−0.24 *	−0.18	−0.10	−0.15
CANTAB RTI Simple Median Reaction Time (ms)	480.06 (159.76)	488.36 (205.65)	−8.31	−0.07	0.80	−0.19	−0.26	−0.05	−0.17	−0.21
CANTAB RTI Simple Median Movement Time (ms)	342.98 (152.42)	335.72 (124.20)	7.26	0.07	0.70	0.15	−0.20	−0.33 *	−0.37 *	−0.38 **
KiTAP Alertness Median Reaction Time (ms)	709.86 (363.35)	661.23 (356.07)	48.63	0.20	0.78	−0.40 **	−0.18	−0.14	−0.12	−0.21

*p < 0.05, ** *p* < 0.01, *** *p* < 0.001; ^a^ Stanford-Binet, fifth edition deviation scores; VABS-3 = Vineland Adaptive Behavior Scale, Third Edition; EVT = Expressive Vocabulary Test; PPVT = Peabody Picture Vocabulary Test; DAS-II = Differential Ability Scales, Second Edition; WISC-V = Wechsler Intelligence Scale for Children, Fifth Edition; Leiter-3 = Leiter International Performance Scale, Third Edition; CANTAB RTI = Cambridge Neuropsychological Test Automated Battery Reaction Time; KiTAP = Test of Attentional Performance for Children.

**Table 3:** Convergent validity correlations at Time 1.

	1	2	3	4	5	6	7
1. DAS-II Rapid Naming Ability Score							
2. WISC-V Cancellation Total Raw Score	0.44 ***						
3. Leiter-3 Attention Sustained Total Raw Score	0.45 ***	0.75 ***					
4. Cat/dog Stroop Congruent Time (s)	−0.67 ***	−0.59 ***	−0.52 ***				
5. Cat/dog Stroop Incongruent Time (s)	−0.66 ***	−0.32 *	−0.41 ***	0.53 ***			
6. CANTAB RTI Simple Median Reaction Time	−0.37 *	−0.39 *	−0.43 **	0.25	0.22		
7. CANTAB RTI Simple Median Movement Time	−0.13	−0.43 **	−0.38 *	0.18	−0.02	0.52 ***	
8. KiTAP Alertness Median Reaction Time	−0.49 ***	−0.56 ***	−0.47 ***	0.45 ***	0.45 **	0.59 ***	0.38 *

* *p* < 0.05, ** *p* < 0.01, *** *p* < 0.001; DAS-II = Differential Ability Scales, Second Edition; WISC-V = Wechsler Intelligence Scale for Children, Fifth Edition; Leiter-3 = Leiter International Performance Scale, Third Edition; CANTAB RTI = Cambridge Neuropsychological Test Automated Battery Reaction Time; KiTAP = Test of Attentional Performance for Children.

**Table 4:** Sensitivity and specificity for examiner-administered processing speed measures below feasibility criterion.

	WISC-V Cancellation				Leiter-3 Attention Sustained				Cat/dog Stroop Incongruent			
	Sensitivity		Specificity		Sensitivity		Specificity		Sensitivity		Specificity	
	Age 8	Age 10	Age 8	Age 10	Age 8	Age 10	Age 8	Age 10	Age 8	Age 10	Age 8	Age 10
No ABIQ Deviation Restriction	97.3%	84.9%	33.3%	41.7%	98.6%	85.7%	33.3%	40.7%	92.2%	80.0%	42.9%	42.9%
ABIQ Deviation ≥ 30	71.2%	58.9%	70.8%	79.2%	74.3%	61.4%	74.1%	81.5%	64.4%	52.2%	85.7%	85.7%
ABIQ Deviation ≥ 40	45.2%	34.3%	87.5%	91.7%	47.1%	35.7%	88.9%	92.6%	40.0%	30.0%	100.0%	100.0%
ABIQ Deviation ≥ 50	21.9%	16.4%	95.8%	95.8%	22.9%	17.1%	96.3%	96.3%	18.9%	14.4%	100.0%	100.0%

WISC-V = Wechsler Intelligence Scale for Children, Fifth Edition; Leiter-3 = Leiter International Performance Scale, Third Edition.

**Table 5:** Sensitivity and specificity for computer-facilitated reaction time measures below feasibility criterion.

	CANTAB RTI				KiTAP Alertness			
	Sensitivity		Specificity		Sensitivity		Specificity	
	Age 8	Age 10	Age 8	Age 10	Age 8	Age 10	Age 8	Age 10
No ABIQ Deviation Restriction	94.2%	90.4%	15.6%	35.6%	95.0%	91.7%	18.9%	43.2%
ABIQ Deviation ≥ 30	71.2%	67.3%	51.1%	71.1%	65.0%	61.7%	46.0%	70.3%
ABIQ Deviation ≥ 40	40.4%	38.5%	66.7%	84.4%	38.3%	35.0%	64.9%	83.8%
ABIQ Deviation ≥ 50	23.1%	21.2%	89.0%	95.6%	20.0%	18.3%	86.5%	94.6%

CANTAB RTI = Cambridge Neuropsychological Test Automated Battery Reaction Time; KiTAP = Test of Attentional Performance for Children.

**Table 6:** Psychometric evaluation summary for processing speed and reaction time measures.

	Minimal Floor Effects	Feasibility	Negligible Practice Effects	Test-Retest	Convergent Validity	
DAS-II Rapid Naming Ability Score	+	+	+	+	Cat/dog Stroop	
DAS-II Rapid Naming T-Score ^a^	+	+	+	+		
WISC-V Cancellation Total Raw Score	−	−	+	+	Leiter-3 Attention Sustained, Cat/dog Stroop (Congruent) & KiTAP Alertness	
WISC-V Cancellation Scaled Score ^a^	−	−	+	+		
Leiter-3 Attention Sustained Total Raw Score	−	−	+	+	WISC-V Cancellation & Cat/dog Stroop (Congruent)	
Leiter-3 Attention Sustained Scaled Score ^a^	−	−	+	+		
Cat/dog Stroop Congruent Time (s)	+	+	+	−	DAS-II Rapid Naming, WISC-V Cancellation, & Leiter-3 Attention Sustained	
Cat/dog Stroop Incongruent Time (s)	−	−	+	−		
CANTAB RTI Simple Median Reaction Time	−	−	+	+	KiTAP Alertness	
CANTAB RTI Simple Median Movement Time	−	−	+	−		
KiTAP Alertness Median Reaction Time	−	−	+	+	WISC-V Cancellation & CANTAB RTI	
Conners CPT-3 T-score ^b^	−	−				

+ Study criterion met: < 20% floor effects, ≥ 80% feasibility, small and not significant practice effects, ICC ≥ 0.75. − Study criterion not met: ≥ 20% floor effects, < 80% feasibility, medium/large and significant practice effects, ICC < 0.75. ^a^ Standard and scaled scores not assessed for convergent validity; ^b^ Conners CPT-3 not assessed for reliability, practice effects, or validity due to exceedingly low feasibility.

## Data Availability

The data that support the findings of this study are available from the corresponding author upon reasonable request.
